# Safety, Pharmacokinetics, and Mosquito‐Lethal Effects of Ivermectin in Combination With Dihydroartemisinin‐Piperaquine and Primaquine in Healthy Adult Thai Subjects

**DOI:** 10.1002/cpt.1716

**Published:** 2019-12-27

**Authors:** Kevin C. Kobylinski, Podjanee Jittamala, Borimas Hanboonkunupakarn, Sasithon Pukrittayakamee, Kanchana Pantuwatana, Siriporn Phasomkusolsil, Silas A. Davidson, Markus Winterberg, Richard M. Hoglund, Mavuto Mukaka, Rob W. van der Pluijm, Arjen Dondorp, Nicholas P.J. Day, Nicholas J. White, Joel Tarning

**Affiliations:** ^1^ Department of Entomology Armed Forces Research Institute of Medical Sciences Bangkok Thailand; ^2^ Entomology Branch Walter Reed Army Institute of Research Silver Spring Maryland USA; ^3^ Department of Tropical Hygiene Faculty of Tropical Medicine Mahidol University Bangkok Thailand; ^4^ Department of Clinical Tropical Medicine Faculty of Tropical Medicine Mahidol University Bangkok Thailand; ^5^ Mahidol‐Oxford Tropical Medicine Research Unit Faculty of Tropical Medicine Mahidol University Bangkok Thailand; ^6^ The Royal Society of Thailand Dusit Bangkok Thailand; ^7^ Centre for Tropical Medicine and Global Health Nuffield Department of Clinical Medicine University of Oxford Oxford UK

## Abstract

Mass administration of antimalarial drugs and ivermectin are being considered as potential accelerators of malaria elimination. The safety, tolerability, pharmacokinetics, and mosquito‐lethal effects of combinations of ivermectin, dihydroartemisinin‐piperaquine, and primaquine were evaluated. Coadministration of ivermectin and dihydroartemisinin‐piperaquine resulted in increased ivermectin concentrations with corresponding increases in mosquito‐lethal effect across all subjects. Exposure to piperaquine was also increased when coadministered with ivermectin, but electrocardiograph QT‐interval prolongation was not increased. One subject had transiently impaired liver function. Ivermectin mosquito‐lethal effect was greater than predicted previously against the major Southeast Asian malaria vectors. Both *Anopheles dirus* and *Anopheles minimus* mosquito mortality was increased substantially (20‐fold and 35‐fold increase, respectively) when feeding on volunteer blood after ivermectin administration compared with *in vitro* ivermectin‐spiked blood. This suggests the presence of ivermectin metabolites that impart mosquito‐lethal effects. Further studies of this combined approach to accelerate malaria elimination are warranted.

**Study Highlights**

**WHAT IS THE CURRENT KNOWLEDGE ON THE TOPIC?**

☑ Ivermectin kills the Anopheline malaria vector and mass drug administration (MDA) can suppress malaria transmission. Ivermectin could be combined with antimalarial drug MDA, but safety evaluations are limited.

**WHAT QUESTION DID THIS STUDY ADDRESS?**

☑ This study assessed the safety, tolerability, pharmacokinetic interaction, and mosquito‐lethal effect of combinations of ivermectin, dihydroartemisinin‐piperaquine, and primaquine in healthy Thai adults.

**WHAT DOES THIS STUDY ADD TO OUR KNOWLEDGE?**

☑ A drug–drug interaction occurred during the coadministration of ivermectin and dihydroartemisinin‐piperaquine, leading to increased ivermectin concentrations and mosquito‐lethal effects, with transaminase rises in one subject. The exposure to piperaquine was increased when co‐administered with ivermectin, but was not associated with increased QT‐interval prolongation. *Anopheles dirus* and *Anopheles minimus* mosquito mortality was substantially increased when fed volunteer blood after ivermectin administration compared with ivermectin‐spiked blood, potentially explained by ivermectin metabolites with mosquito‐lethal effects.

**HOW MIGHT THIS CHANGE CLINICAL PHARMACOLOGY OR TRANSLATIONAL SCIENCE?**

☑ There may be slowly eliminated ivermectin metabolites with mosquito‐lethal activities. Ivermectin plus dihydroartemisinin‐piperaquine MDA could be used for malaria elimination but potential for hepatotoxicity needs further study.

Novel control and elimination measures are needed urgently to contain artemisinin and partner drug resistance in the Greater Mekong Subregion (GMS). With new drugs still years away, it is proposed that this can be achieved only by elimination of *Plasmodium falciparum* from the region.^1^ In recent years, mass drug administrations (MDAs) using the artemisinin‐based combination therapy (ACT) dihydroartemisinin‐piperaquine with primaquine have been evaluated throughout the GMS.^2^, ^3^ Dihydroartemisinin rapidly clears *Plasmodium*‐infected individuals of the majority of their asexual parasites, and piperaquine eliminates the residuum to prevent recrudescent infections while providing post‐treatment prophylaxis for ~ 1 month, which prevents new blood stage infections.^6^, ^7^ Single low‐dose primaquine is added to kill late‐stage gametocytes and prevent onward transmission from infected individuals to *Anopheles* mosquitoes.^8^, ^9^ However, none of these drugs affects infection rates in the extant, already infected *Anopheles* population,^11^ which will continue to transmit malaria parasites.

*Anopheles* mosquitos in the GMS typically feed outdoors, thereby evading classic control efforts, insecticide‐treated bed nets, and indoor residual spraying with insecticides.^12^, ^13^ *In vitro* assays demonstrate that ivermectin, at human‐relevant concentrations, is lethal to the two primary malaria vectors in the GMS, *Anopheles dirus* and *Anopheles minimus*.^15^ Because ivermectin kills mosquitoes following the point of human‐vector contact, ivermectin MDA is a novel vector control tool that targets outdoor malaria transmission directly. In West Africa, ivermectin MDA has been shown to kill wild *Anopheles*, shift the mosquito population age structure, interrupt mosquito transmission,^16^ and reduce clinical incidence of *P. falciparum*.^17^

Combining ivermectin with antimalarial MDA would be a novel way to reduce transmission of *Plasmodium* parasites, as it targets the malaria parasite in the host and mosquito vector. MDA coverage rarely reaches 100% of the eligible population, adherence may be poor, and malaria asymptomatic or healthy people may not perceive a direct personal benefit from participation in ACT‐based MDAs.^18^ However, experience in Africa shows that people do recognize direct personal benefits from participating in ivermectin MDAs because the drug affects several neglected tropical diseases,^19^ which are common in malaria‐endemic areas.^20^ Thus, combining ivermectin with ACT MDAs may both improve overall health outcomes and MDA acceptability. This trial assessed the safety, tolerability, pharmacokinetic interactions, and mosquito‐lethal effect of combinations of ivermectin, dihydroartemisinin‐piperaquine, and primaquine in healthy adult Thai subjects.

## Materials And Methods

### Study design

The study was an open label, single dose, sequential trial, with oral administration of ivermectin, dihydroartemisinin‐piperaquine, and primaquine, alone and in combination. Seven drug regimens were evaluated: ivermectin (400 µg/kg) alone, dihydroartemisinin‐piperaquine (120/960 mg) alone, primaquine (30 mg) alone, dihydroartemisinin‐piperaquine plus primaquine, ivermectin plus primaquine, ivermectin plus dihydroartemisinin‐piperaquine, and ivermectin plus dihydroartemisinin‐piperaquine plus primaquine. Sixteen healthy adult male and female Thai subjects between 18 and 60 years of age were recruited. The sample size was based on prolongation of the QT‐interval. See **Text** **S1** for further detail.

### Safety analysis

Safety was analyzed based on adverse events (AEs), physical examination, vital signs, clinical laboratory parameters, 12‐lead electrocardiogram findings, and methemoglobin levels. See **Text** **S1** for further details.

### Pharmacokinetic analysis

Blood samples were collected at 0 (predose), 0.5, 1, 1.5, 2, 3, 4, 6, 8, 12, and 24 hours and on days 2, 3, 6, 10, 14, 21, and 35. All drug analyses were performed using validated high‐performance liquid chromatography linked with tandem mass spectrometry.^21^, ^22^ Individual subject drug concentration‐time data were evaluated using a noncompartmental approach. Pharmacokinetic parameter estimates from the different regimens were analyzed using a bioequivalence function (i.e., log‐transformed pharmacokinetic exposure parameters peak plasma concentration (C_max_) area under the concentration‐time curve from time zero to the last measured concentration (AUC_T_) and time to maximum concentration (T_max_)) were used to assess the drug when administered alone vs. that in combination. See **Text** **S1** for further details.

### Mosquito survival analyses

*Anopheles dirus* s.s. and *An. minimus* s.s. were reared as described previously.^23^ Whole blood samples were collected in sodium heparin tubes at 0, 4, and 24 hours, and on days 2, 3, 6, and 10 from all drug regimens that contained ivermectin, and blood fed to mosquitoes. See **Text** **S1** for further details.

## Results

### Subjects

Sixteen healthy subjects were enrolled in the study (7 men and 9 women). At baseline, women had significantly higher body mass index, whereas men had significantly higher hemoglobin, albumin, and serum creatinine levels (**Table** 1). All the volunteers completed the study protocol and were included in the safety, mosquito survival, and pharmacokinetic analyses.

**Table 1:** Baseline demographics of study participants

Demographics	Male (*n* = 7)	Female (*n* = 9)
Age (year)	32 (29, 36)	37 (32, 48)
Weight (kg)	62.9 (56.9, 69.8)	62.9 (56.6, 72.8)
Body mass index (kg/m^2^)^a^	21.5 (20.0, 22.3)	23.3 (23.1, 27.6)
Hemoglobin (g/dL)^a^	14.4 (13.7, 15)	12 (11.5, 12.6)
Creatinine (mg/dL)^a^	0.87 (0.8, 0.93)	0.63 (0.62, 0.75)
Albumin (g/dL)^a^	4.6 (4.5, 4.6)	4.3 (4.2, 4.4)
Aspartate transaminase (IU/L)	21 (17, 23)	15 (15, 17)
Alkaline phosphatase (IU/L)	18 (16, 19)	12 (11, 14)
Alkaline phosphatase (IU/L)	64 (48, 89)	57 (50, 71)
Total bilirubin (mg/dL)	0.7 (0.6, 0.9)	0.4 (0.3, 0.5)
QTcF interval (ms)	411 (407, 426)	414 (411, 416)

Data are reported as median (interquartile range) unless otherwise specified.

IU/L, international units per liter; QTcF, Fridericia‐corrected QT‐interval.

a Significant differences (*P* < 0.01) in baseline characteristics between male and female volunteers.

### Safety analysis

The drugs were well‐tolerated. No clinically significant changes in the physical examination or vital signs were observed during the course of the study. All subjects had methemoglobin levels below 2.1% at all times during the study.

**Figure 1:**
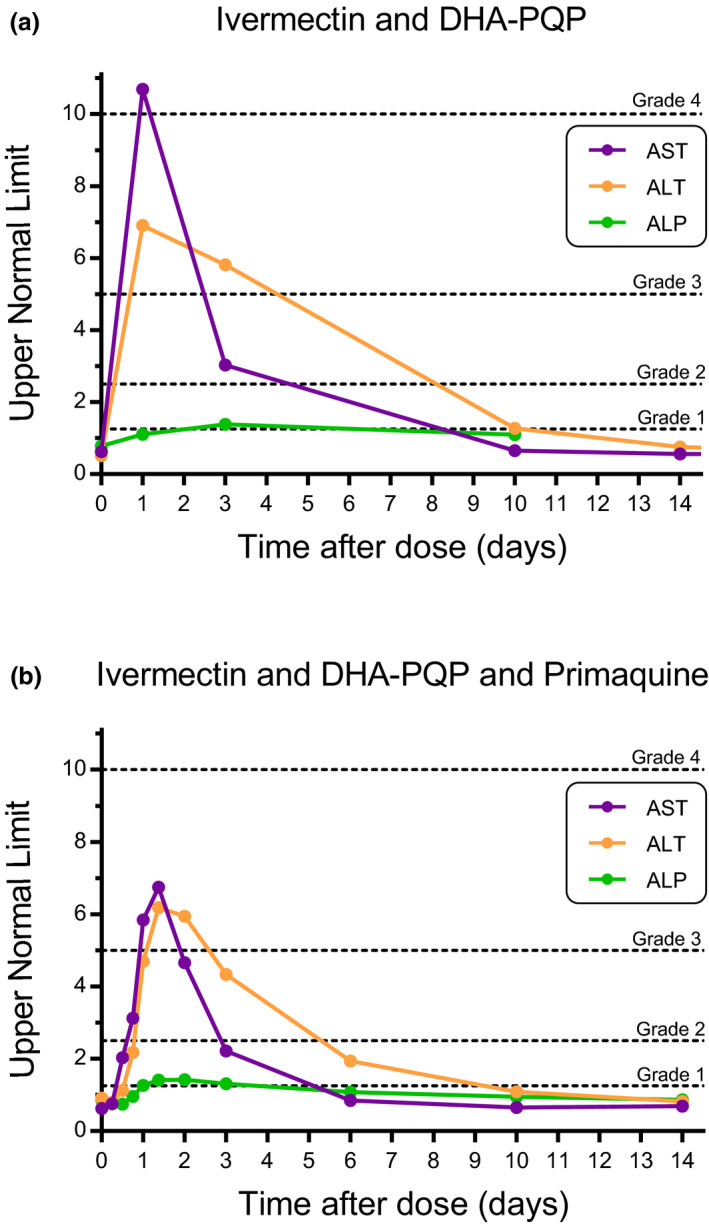
Liver enzymes associated with drug administration of ivermectin, dihydroartemisinin‐piperaquine, and primaquine in one subject. Aspartate transaminase (AST), alanine aminotransferase (ALT), and alkaline phosphatase (ALP) values in one female volunteer following administration of (**a**) ivermectin plus dihydroartemisinin‐piperaquine (DHA‐PQP) and (**b**) ivermectin plus DHA‐PQP plus primaquine. Dashed lines demarcate adverse events grading scale for female subjects at Mahidol Hospital for Tropical Diseases.

Two severe adverse events were reported in two subjects. One male subject was hospitalized due to dengue infection, considered unrelated to the study drug or procedures. One 40‐year‐old female subject had transient elevation of liver enzymes when given ivermectin plus dihydroartemisinin‐piperaquine; her aspartate aminotransferase (AST) levels rose 10.7‐fold (grade 4) above the upper limit of normal (ULN), deemed a severe adverse event by Division of Acquired Immunodeficiency Syndrome toxicity grading,^24^ her alanine transaminase (ALT) rose 6.9‐fold above the ULN (grade 3) and alkaline phosphatase (ALP) 1.4‐fold above the ULN (grade 1). Following ivermectin plus dihydroartemisinin‐piperaquine plus primaquine administration her AST rose 6.8‐fold above the ULN (grade 3), her ALT was 6.2‐fold above the ULN (grade 3), and ALP was 1.4‐fold above the ULN (grade 1; **Figure** 1). This was considered linked to study drug combinations. All values returned to normal within 10 days (**Figure** 1). There was no increase in total bilirubin and so no violation of Hy's Law. She had taken no concomitant medication, was negative for hepatitis A/B/C, had a normal serum lipid profile, and normal hepatobiliary ultrasound. Four other AEs (grade 1) related to liver function tests were observed in other volunteers but each was detected before drug administrations and so considered unrelated to study medications (**Figure** **S1**). For all other volunteers, there were no significant differences in mean rise in liver function test values from baseline (0 hour) to 24 hours post‐drug administration between treatment regimens for AST (*F* value = 0.95; *P* = 0.4630), ALT (*F* value = 1.14; *P* = 0.3428), ALP (*F* value = 0.27; *P* = 0.9507), direct bilirubin (*F* value = 0.67; *P* = 0.6731), or total bilirubin (*F* value = 0.8; *P* = 0.5692; **Figure** **S1**).

During the study, 51 nonhepatobiliary AEs were reported by 14 subjects and all were considered unrelated to the study drugs. The most common other AEs were infections, mostly acute febrile illnesses and common colds (**Table** **S1**). All AEs resolved completely.

**Figure 2:**
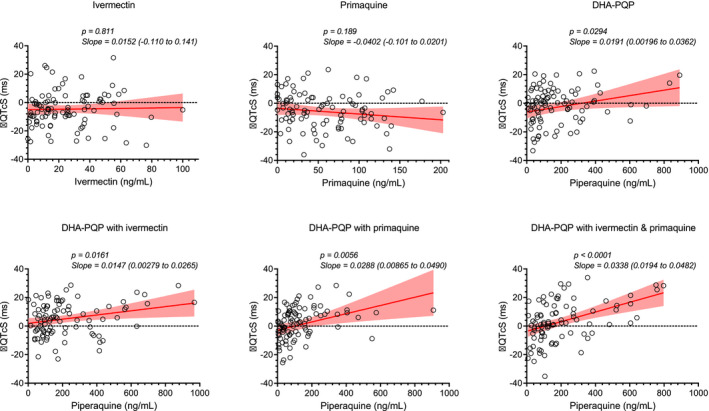
Electrocardiographic effects, stratified by treatment regimen. Open circles represent observed study‐corrected QT‐interval prolongations (ΔQTcS) and associated measured drug concentrations at those particular time points. Solid red lines represent the mean regression line and the shaded areas represent the 95% confidence interval of the mean slope. Dashed black lines represent zero ΔQTcS effect. DHA‐PQP, dihydroartemisinin‐piperaquine.

Observed QT‐intervals were correlated significantly with heart rate. The commonly used Fridericia‐correction (correction factor of 0.333) and Bazett‐correction (correction factor of 0.500) did not resolve this correlation fully (**Figure** **S2**). Thus, all QT measurements and corresponding heart rates were used to estimate an optimal study‐specific correction factor, resulting in an optimal correction factor of 0.428. No subject developed a prolongation of study‐specific corrected QT‐intervals (QTcS) of >60 ms or had an absolute value above 500 ms, two criteria often used to indicate an increased risk of Torsade des Pointes. There was a significant correlation between ∆QTcS and piperaquine concentrations in all piperaquine cohorts but no significant relationship with ivermectin or primaquine concentrations (**Figure** 2). Administration of dihydroartemisinin‐piperaquine alone resulted in a 2.94 (95% confidence interval (CI) 1.91–3.62) millisecond prolongation of QTcS‐intervals per 100 ng/mL increase in piperaquine plasma concentrations. Coadministration of ivermectin and/or primaquine did not augment this (**Figure** 2). There were no significant differences in maximum ∆QTcS between piperaquine groups using a paired analysis of variance (ANOVA; *P* = 0.2358).

### Pharmacokinetic analysis

**Figure 3:**
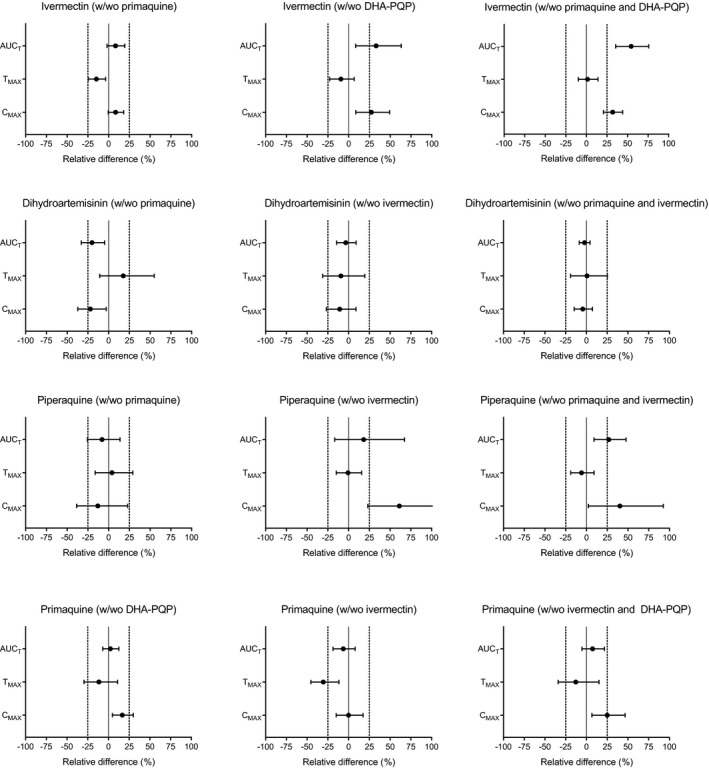
Drug–drug interaction effects, stratified by drug and treatment regimen. Graphs represent Forest plots of geometric mean ratios of pharmacokinetic parameters (circles) and associated 90% confidence intervals around these ratios (bars). The solid vertical lines represent no interaction, whereas vertical dashed lines represent an effect of ± 25% relative difference, deemed to represent a clinical relevant effect. AUC_T_, area under the concentration‐time curve from time zero to the last measured concentration; C_max_, peak plasma concentration; DHA‐PQP, dihydroartemisinin‐piperaquine; T_max_, time to reach maximum concentration.

There was no significant difference in ivermectin exposure between male and female subjects (*P*> 0.05). All drug–drug interactions are illustrated in **Figure** 3 and summarized in **Table** **S2**. Pharmacokinetic parameter estimates, stratified by treatment regimen, are summarized in **Table** **S3**. In brief, coadministration of dihydroartemisinin‐piperaquine and ivermectin resulted in a significant increase in peak concentrations and overall exposure to ivermectin (C_max_: 27.3%; AUC_T_: 33.1%). This drug–drug interaction was augmented when ivermectin was given with both dihydroartemisinin‐piperaquine and primaquine (C_max_: 31.9%; AUC_T_: 54.4%). Coadministration with primaquine had no significant impact on the pharmacokinetic properties of ivermectin, other than a small but clinically insignificant reduction in the time to peak concentrations (T_max_: −14.7%).

The pharmacokinetic properties of piperaquine were influenced by coadministration of ivermectin, resulting in substantially increased peak concentrations of piperaquine (C_max_: 61.1%). Coadministration of primaquine had no effect on the pharmacokinetic properties of piperaquine, but coadministration of both primaquine and ivermectin significantly increased peak piperaquine concentrations and overall exposure (C_max_: 40.3%; AUC_T_: 26.9%).

The pharmacokinetic properties of dihydroartemisinin were not affected by coadministration of ivermectin, or both ivermectin and primaquine. However, coadministration of primaquine alone slightly reduced peak dihydroartemisinin concentrations and overall exposure (C_max_: 21.9%, AUC_T_: −20.1%).

No significant impact was seen on the pharmacokinetic properties of primaquine when co‐administered with ivermectin, except a reduction in the time to peak concentrations (T_max_: −30.5%). Peak primaquine concentrations were slightly increased with coadministration of dihydroartemisinin‐piperaquine (C_max_: 16.7%) and with both dihydroartemisinin‐piperaquine and ivermectin (C_max_: 25.0%).

Drug measurements of ivermectin in venous and capillary blood showed an ~1:1 correlation with no substantial difference between male (slope = 0.962; 90% CI 0.910–1.014) and female (slope = 1.073; 90% CI 1.017–1.130) subjects (**Figure** **S3** **a**). This 1:1 correlation was maintained when comparing between ivermectin alone (slope = 1.037; 90% CI 0.997–1.078) and ivermectin plus dihydroartemisinin‐piperaquine (slope = 0.999; 90% CI 0.936–1.063; **Figure** **S3** **b**). Two female subjects had detectable levels of ivermectin in capillary blood at 56 days (1.12 ng/mL) and at 69 days (1.48 ng/mL) after ivermectin plus primaquine administration.

### Mosquito survival analysis

**Figure 4:**
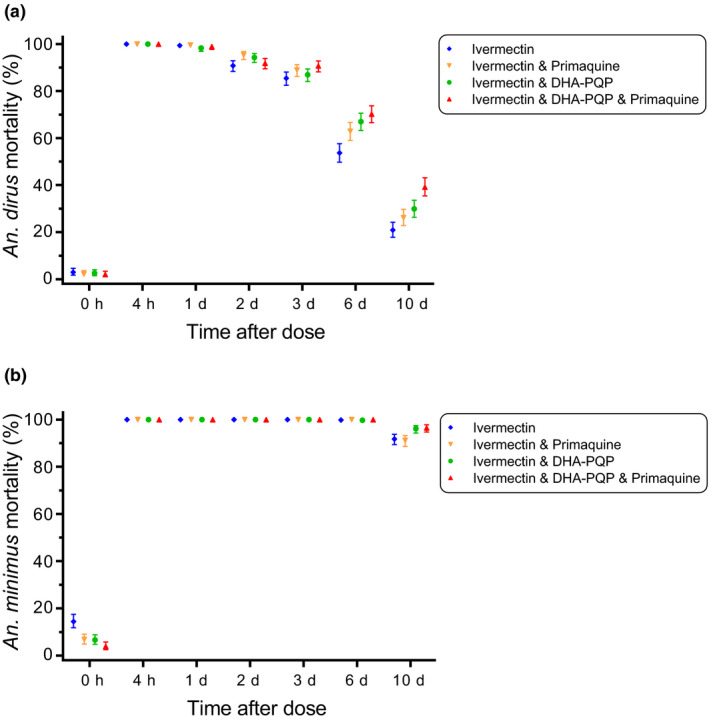
Mosquito lethal effects of ivermectin, across all drug regimens, stratified by *Anopheles dirus* (**a**) and *Anopheles minimus* (**b**). Filled symbols represent the cumulative mean mosquito mortality at 10 days post‐blood meal, across the different ivermectin‐containing drug regimens, at each blood collection time point. Bars represent the 95% confidence intervals around these means. DHA‐PQP, dihydroartemisinin‐piperaquine.

**Figure 5:**
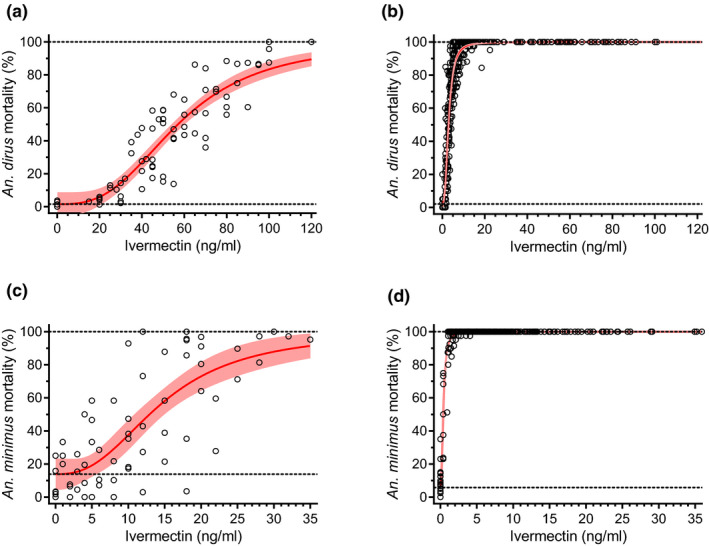
Mosquito lethal effects of ivermectin, after membrane feeding to *Anopheles dirus* (**a, b**) and *Anopheles minimus* (**c, d**), using ivermectin‐spiked blood **a, c** and human volunteer blood after ivermectin administration **b, d**. *In vitro* spiked blood **a, c** represents healthy human blood spiked with known concentrations of ivermectin reference standard. *In vivo* volunteer blood **b, d** represents human volunteer blood collected in healthy volunteers at various time points after oral administration of ivermectin (400 μg/kg), and drug concentrations measured using liquid chromatography mass spectrometry. Open circles represent cumulative mosquito mortality 7 days after blood meal ingestion. Solid red lines represent the mean concentration‐response relationship and the dashed red lines represent the 95% confidence interval associated with the nonlinear fit. Dashed black lines represent the estimated minimum effects based on control mosquito mortality, and fixed maximum effects of 100% mortality.

**Figure 6:**
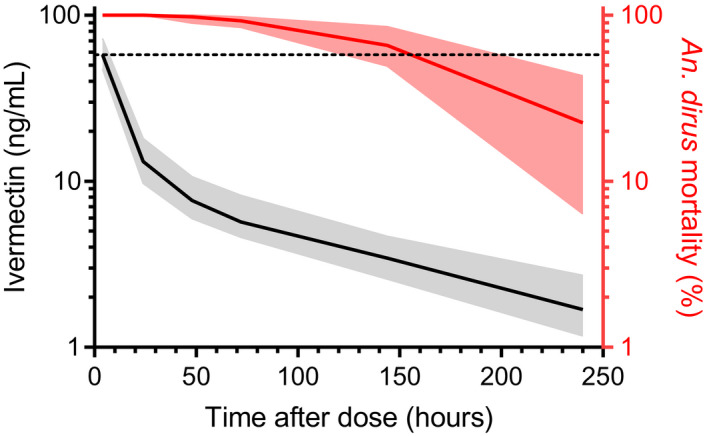
Pharmacokinetic and pharmacodynamic effects of ivermectin. Observed ivermectin concentration (black, left *y*‐axis) and *Anopheles dirus* mosquito mortality (red; right *y*‐axis) plotted vs. time. Solid lines represent median values and shaded areas represent the interquartile range (25–75th percentile). The dashed black line represents the *An. dirus in vitro* 7‐day‐lethal concentration 50%‐value (57.65 ng/mL).

A total of 17,946 *An. dirus* and 17,626 *An. minimus* were evaluated in survival assays. Survival of both *An. dirus* and *An. minimus* was reduced significantly (*P* < 0.0001) when fed volunteer blood collected at all time points, compared with baseline controls (hour 0), across all treatment regimens (**Figure** **S4**). There was significantly increased mosquito mortality for *An. dirus* when fed volunteer blood from days 6 and 10 (**Figure** 4 **a**) and for *An. minimus* when fed volunteer blood from day 10 (**Figure** 4 **b**) in all regimens containing dihydroartemisinin‐piperaquine compared with regimens of ivermectin alone. For *An. dirus*, mosquito mortality was increased by 20.2‐fold when fed human volunteer blood after ivermectin administration (*in vivo* 7‐day lethal concentration 50% (LC_50_) = 2.86 ng/mL; 95% CI 2.67–3.05; **Figure** 5 **b**) compared with our previous *in vitro* results with ivermectin compound mixed with human blood (*in vitro* 7‐day‐LC_50_ = 57.65 ng/mL; 95% CI 52.30–63.12; **Figure** 5 **a**).^15^ For *An. minimus*, mosquito mortality was 35.0‐fold higher when fed human volunteer blood after ivermectin administration (*in vivo* 7‐day‐LC_50_ = 0.42 ng/mL; 95% CI 0.39–0.46; **Figure** 5 **d**) compared with our previous *in vitro* results (7‐day‐LC_50_ = 14.68 ng/mL; 95% CI 11.51–18.12; **Figure** 5 **c**).^15^ Comparing the ivermectin concentration‐time curve and the *An. dirus* mortality‐time curve illustrates the discordance between pharmacokinetic and pharmacodynamic processes based on parent compound concentrations alone, suggesting the presence of more slowly eliminated ivermectin metabolites with mosquito‐lethal effects (**Figure** 6).

There were small but significant differences in *An. dirus* mortality when using human volunteer blood from men (10‐day‐LC_50_ = 2.35 ng/mL; 95% CI 2.23–2.48) and women (10‐day‐LC_50_ = 2.90 ng/mL; 95% CI 2.75–3.04). There were also minor differences in *An. dirus* mortality between drug regimens: ivermectin alone (10‐day‐LC_50_ = 2.66 ng/mL; 95% CI 2.49–2.83), ivermectin plus primaquine (10‐day‐LC_50_ = 2.53 ng/mL; 95% CI 2.24–2.83), ivermectin plus dihydroartemisinin‐piperaquine (10‐day‐LC_50_ = 2.67 ng/mL; 95% CI 2.46–2.89), and ivermectin plus dihydroartemisinin‐piperaquine plus primaquine (10‐day‐LC_50_ = 2.95 ng/mL; 95% CI 2.75–3.15). The estimated intracluster correlation value for *An. dirus* mortality was 0.264. Mortality rates in *An. minimus* were extremely high (**Figure** 4, **Figure** **S4**) with 10‐day‐LC_50_ values below the lower limit of ivermectin detection (0.776 ng/mL), therefore, further analyses of *An. minimus* mortality were not performed. Hazard ratios for mosquito mortality by day post‐blood meal ingestion indicate that most mosquito mortality occurs in the first 3 days for both species. Blood from later time points post–drug administration (e.g., days 6 and 10) fed to *An. dirus* had a delayed lethal effect. Hazard ratios for mortality for *An. dirus* and *An. minimus* (**Figure** **S5**) were much higher than for *An. gambiae*,^25^ but this was due in part to the high baseline control (hour 0) survival (**Figure** 4, **Figure** **S4**). *In vitro* membrane feeding results with piperaquine‐spiked blood indicated no effect of parent compound on *An. dirus* survival (*P* > 0.05; **Figure** **S6**).

## Discussion

All drugs were well tolerated. The only apparent safety concern was a presumed drug–drug interaction in a 40‐year‐old female subject, which occurred on both occasions when ivermectin was co‐administered with dihydroartemisinin‐piperaquine. Her AST and ALT levels rose quickly postdose but returned to normal within 10 days (**Figure** 1) without a concomitant rise in bilirubin (i.e., no violation of Hy's Law) and without symptoms. Further investigation revealed no alternative cause for these increases in liver enzymes, and the ivermectin and piperaquine concentrations were not elevated compared with other volunteers.

Piperaquine has been rarely associated with liver injury. A multicountry study in Africa with dihydroartemisinin‐piperaquine treatment of > 10,000 malaria‐infected patients found elevated levels of AST and ALT (> 2 × ULN) on day 7, in 19 and 13 patients, respectively.^26^ It is unclear whether those elevations were related to malaria or to its treatment. One case of mild hepatitis was linked to dihydroartemisinin‐piperaquine in a *P. falciparum*‐infected patient.^27^ There are some reports of hepatotoxicity associated with ivermectin. A previous trial reported an increased ALT and gamma‐glutamyl transferase, possibly linked to ivermectin, in a 37‐year‐old woman with cholelithiasis after ivermectin treatment with a dose of 2 mg/kg, which is five times higher than doses used in this trial.^28^ Minor increases in AST and ALT were observed in three elderly Japanese patients when treated with ivermectin (200 µg/kg twice 1 week apart) for scabies who were also on zopiclone, amlopidine, lansoprazole, zolpidem, and rabeprazole.^29^ There are two reports of ivermectin alone inducing liver damage, resulting in hepatitis in a 20‐year‐old female with *Loa loa* 30 and prolonged liver dysfunction in an 85‐year‐old man with scabies.^31^ In a previously published trial, 3 of 90 patients with *falciparum* who received ivermectin (300 or 600 µg/kg) and dihydroartemisinin‐piperaquine for 3 consecutive days had elevated AST and/or ALT values (grade 3). Two patients had grade 1 levels and one patient had grade 3 levels at enrollment.^25^ The findings of increased AST and ALT following ivermectin and dihydroartemisinin‐piperaquine in the current and previous^25^ trials warrant further investigation.

The ivermectin plus primaquine (30 mg) combination was well tolerated and no AEs were reported for this combination. This combination could be considered for MDAs to provide simultaneous radical cure of *Plasmodium vivax* and to prevent malaria transmission.^32^ Further evaluation of ivermectin plus primaquine safety in *P. vivax*–infected G6PD deficient persons is warranted before full scale MDAs occur.

The overall exposure to ivermectin was increased substantially by concomitant administration of dihydroartemisinin‐piperaquine (increase of 33%), which was augmented further when primaquine was added (increase of 54%). The mechanism of this drug–drug interaction is unclear, but could be a consequence of interaction at CYP3A4, the main metabolic pathway for both ivermectin^33^ and piperaquine.^34^ Coadministration of primaquine alone did not have a significant impact on the exposure to ivermectin, suggesting that the drug–drug interaction is driven primarily by dihydroartemisinin‐piperaquine. Ivermectin is safe in much larger doses^28^ than administered here, so this increase in ivermectin exposure is unlikely to be of clinical significance.

The pharmacokinetic properties of piperaquine were unaffected when primaquine alone was co‐administered. Although peak piperaquine concentrations were elevated when co‐administered with ivermectin (a 61% increase) there was no impact on total exposure. However, ivermectin plus primaquine did increase exposure to piperaquine, with a 27% and 40% increase in total exposure and peak concentrations, respectively. This could be a consequence of a competition for CYP3A4 biotransformation. In contrast, a previous pharmacokinetic study showed that piperaquine parameter estimates were not influenced by coadministration of ivermectin.^35^ Possible explanations for these differences between studies could relate to the different study designs, populations (Thai and Kenyan) and disease states (healthy and *P. falciparum* infected).

The substantial increases in peak plasma concentrations of piperaquine when co‐administered with ivermectin alone (61%) or with primaquine (40%), did not have a significant effect on QT‐interval prolongation (**Figure** 2), suggesting that these pharmacokinetic interactions did not translate into an increased risk of arrhythmia. Furthermore, the overall relationship between piperaquine concentrations and ∆QTcS was not substantially different between regimens. However, this study was not designed as a thorough QT study. Thus, one limitation is that no placebo regimen was included, and it is, therefore, not possible to apply double‐delta corrections, and compensate for potential circadian changes in heart rate and/or QT‐intervals.

The pharmacokinetic properties of primaquine were largely unaffected by coadministration of ivermectin. A previous study demonstrated a significant reduction in primaquine clearance and volume of distribution, resulting in higher peak concentrations and increased overall exposure, when co‐administered with dihydroartemisinin‐piperaquine.^36^ This study shows the same trend of increased peak concentrations both when given together with dihydroartemisinin‐piperaquine alone or with ivermectin plus dihydroartemisinin‐piperaquine. This is unlikely to be of clinical significance.

As expected, the pharmacokinetic properties of dihydroartemisinin were unaffected by concomitant administration of ivermectin and/or primaquine, except for a small but probably clinically insignificant reduction in peak concentrations (22%) when co‐administered with primaquine alone, but total exposure was unaffected.

Ivermectin was lethal to *An. dirus* and *An. minimus* (**Figure** 4 and **Figure** **S4**) for a much longer time than predicted previously from a pharmacokinetic‐pharmacodynamic model linking *in vitro* membrane feeding results with ivermectin compound spiked in human blood.^15^ Ivermectin 7‐day‐LC_50_‐values for *An. dirus* and *An. minimus* from this trial were 20‐fold and 35‐fold lower than the *in vitro* results, respectively (**Figure** 5). There was also a substantial temporal discrepancy when comparing ivermectin plasma concentration‐time data with mosquito‐lethal effects over time. Ivermectin concentrations fell below the estimated *in vitro* 7‐day‐LC_50_ within 24 hours of drug administration, whereas more than half of the maximum mosquito‐killing effect was maintained for up to 7 days after drug administration (**Figure** 6). One possible explanation is that previously uncharacterized, slowly eliminated ivermectin metabolites may have mosquito‐lethal effects, increasing mosquito mortality beyond that predicted by the parent compound alone. Some 10–12 ivermectin metabolites have been identified using animal and human liver microsomes.^33^, ^37^ A very small (*n* = 4) ivermectin mass balance study determined that mean peak plasma concentrations of these metabolites were 2.5‐fold higher than that of the parent compound, and the effective half‐lives of the metabolites were approximately 6‐fold longer compared with ivermectin.^38^

In this and previous studies at this location, all mosquito experiments were performed at the same facility, with the same mosquito strains, and both *in vitro* results (membrane feeding mosquitoes with blood spiked with ivermectin) and *in vivo* results (membrane feeding mosquitoes with blood from human volunteers receiving ivermectin) showed close to 100% survival in control mosquitoes. Thus, active ivermectin metabolites, with mosquito‐lethal properties, seem a plausible explanation to this substantially (20‐fold and 35‐fold) increased susceptibility to ivermectin *in vivo*. If slowly eliminated ivermectin metabolites impart a mosquito‐lethal effect, then this would explain the longer duration of mosquito mortality than initially predicted.^15^ This greater than previously estimated period of mosquito killing supports the use of ivermectin MDA for malaria transmission suppression in the GMS.

When ivermectin was co‐administered with dihydroartemisinin‐piperaquine there was an increase in *An. dirus* mortality compared with ivermectin alone when the mosquitoes fed blood from days 6 and 10 postdose (**Figure** 4 **a**), and an increase in *An. minimus* mortality when fed blood from day 10 postdose (**Figure** 4 **b**). *In vitro* membrane feeding results with piperaquine‐spiked blood indicated no effect of parent compound on *An. dirus* survival (**Figure** **S6**). Furthermore, if dihydroartemisinin‐piperaquine was lethal to mosquitoes, it would have reduced *An. dirus* ivermectin LC_50_‐values associated with dihydroartemisinin‐piperaquine‐containing regimens below that of ivermectin alone, but this did not occur. Coadministration of ivermectin with dihydroartemisinin‐piperaquine led to increased C_max_ and AUC_T_ values for ivermectin (**Figure** 3 **a**), which presumably explains the increased mosquito mortality with dihydroartemisinin‐piperaquine‐containing regimens (**Figure** 4), and may also explain the prolonged mosquito‐lethal effect observed with *An. gambiae* in a previous trial.^25^ An effect on bioactive ivermectin metabolite disposition may also occur.

Blood from male subjects was slightly more lethal to *An. dirus* than from female subjects. This contradicts findings from two previous studies in *P. falciparum*‐infected patients in which ivermectin was co‐administered with artemether‐lumefantrine in Burkina Faso^39^ and dihydroartemisinin‐piperaquine in Kenya.^25^ However, this discrepancy could be an artifact of small sample size in the current study. As both male and female subjects’ blood was lethal to *An. dirus* with ivermectin 10‐day‐LC_50_‐values below 3 ng/mL, it is unlikely that this difference would be of major relevance in field MDA settings.

Capillary and venous blood ivermectin concentrations were similar (**Figure** **S3**). Ivermectin is a very lipophilic drug, which disperses into dermal compartments at twofold to threefold higher concentrations than venous plasma in both rats^40^ and humans.^41^ Mosquitoes feed from subdermal capillaries and not primary veins and arteries. It was proposed previously that mosquitoes may imbibe higher ivermectin concentrations than predicted from venous plasma.^42^ It is also possible that ivermectin metabolites may occur at higher concentrations in subdermal capillaries and this could contribute to enhanced mosquito‐lethal effects. Two previous studies showed conflicting results. In a study from Brazil, ivermectin was more lethal to *An. aquasalis* that directly fed on ivermectin‐treated persons compared with membrane‐fed venous blood from the same volunteers,^43^ whereas no difference was observed for *An. gambiae* in Kenya with similar experimental design.^44^ The detection of ivermectin in capillary blood in two subjects at 56 and 69 days postdose of ivermectin plus primaquine suggest a possible very slow terminal elimination phase. Further comparisons of membrane feeds with venous blood and direct mosquito feeds should be conducted over protracted periods.

MDAs with ACTs are unlikely to select for drug resistance in asymptomatic persons.^45^ The addition of ivermectin during dihydroartemisinin‐piperaquine MDA could further reduce likelihood of antimalarial drug resistance development and spread by several mechanisms. Increased piperaquine concentrations during ivermectin coadministration reduce the likelihood of parasite exposure to suboptimal partner drug concentrations. Conversely, if suboptimal piperaquine concentrations occur due to incomplete ACT treatment, missed doses, poor absorption, or unusual pharmacokinetics, then the enhanced reduction in parasite transmission by ivermectin would reduce the number of new infections exposed to suboptimal partner drug concentrations. Finally, drug‐resistant parasites ingested by mosquitoes before MDA or from untreated persons would not be transmitted if the mosquito is killed by ivermectin during a later blood meal before the mosquito has developed infectious sporozoites.

This study found the combination of ivermectin plus dihydroartemisinin‐piperaquine plus primaquine to be generally well tolerated. One subject had a drug–drug interaction with ivermectin plus dihydroartemisinin‐piperaquine, which increased AST and ALT to levels of concern and warrants further investigation. Coadministration of ivermectin plus dihydroartemisinin‐piperaquine led to increased concentrations of ivermectin, which imparted a greater mosquito‐lethal effect. The much greater mosquito‐lethal effect demonstrated in this study compared with previous ivermectin *in vitro* results suggest that there may be ivermectin metabolites with mosquito‐lethal effects.

## Disclaimer

Material has been reviewed by the Walter Reed Army Institute of Research. There is no objection to its presentation and/or publication. The opinions or assertions contained herein are the private views of the authors, and are not to be construed as official, or as reflecting true views of the Department of the Army or the Department of Defense. The investigators have adhered to the policies for protection of human subjects as prescribed in AR 70–25.

## Funding

This study was funded by the UK Department for International Development (GB‐1‐201900). The Mahidol Oxford Tropical Medicine Research Unit is funded by the Wellcome Trust of Great Britain. The pharmacokinetic work was funded through a grant from the Bill & Melinda Gates Foundation (OPP1134284). The funders had no part in the study design, implementation, or analysis of the study or in the decision to publish the results.

## Conflict of Interest

The authors declared no competing interests for this work.

## Author Contributions

K.C.K., P.J., R.vdP., R.H., N.J.W., and J.T. wrote the manuscript. K.C.K., P.J., B.H., R.vdP., A.D., N.J.W., and J.T. designed the research. K.C.K., P.J., B.H., and K.P. performed the research. K.C.K., P.J., M.W., R.M.H., M.M., and J.T. analyzed the data.

## Supporting information


**Figure S1.**
**Figure S2.**
**Figure S3.**
**Figure S4.**
**Figure S5.**
**Figure S6.**
**Table S1.**
**Table S2.**
**Table S3.**
**Supplemental Text, Figure, and Table Legends.**
