# Supplementary material for: Safety, Pharmacokinetics, and Mosquito‐Lethal Effects of Ivermectin in Combination With Dihydroartemisinin‐Piperaquine and Primaquine in Healthy Adult Thai Subjects
Source: Clin Pharmacol Ther. 2019 Dec 27;107(5):1221–30. doi: 10.1002/cpt.1716 (PMC7285759; doi:10.1002/cpt.1716)
Supplement: Supplementary file 1 — Figure S1. Figure S2. Figure S3. Figure S4. Figure S5. Figure S6. Table S1. Table S2. Table S3. Supplemental Text, Figure, and Table Legends. [file CPT-107-1221-s001.pdf]

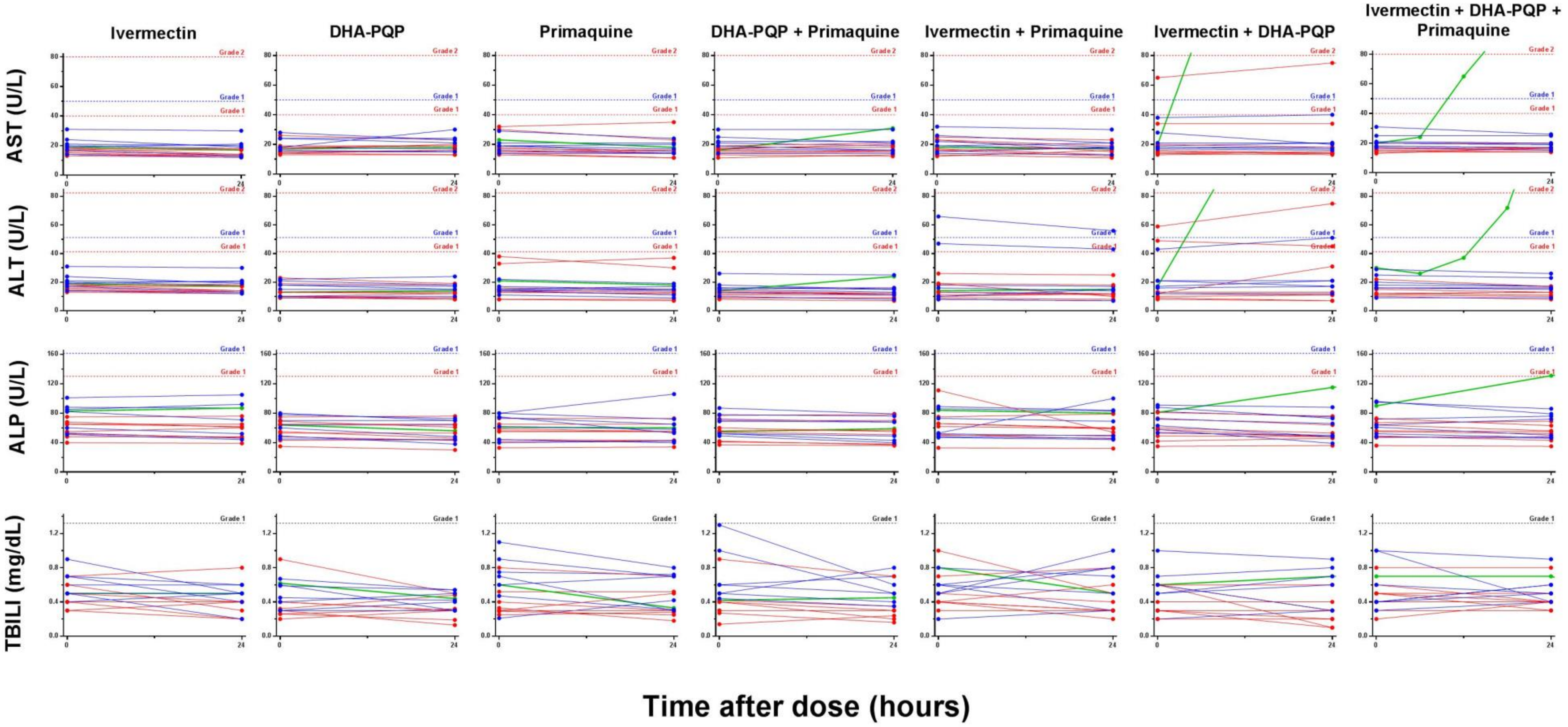

Figure S1. Liver function test results for all subjects, stratified by treatment regimen.

Aspartate transaminase (AST), alanine aminotransferase (ALT), alkaline phosphatase (ALP), and total bilirubin (TBILI) values in all subjects following administration of ivermectin, dihydroartemisinin-piperaquine (DHA-PQP), and primaquine alone or in various combinations. Male (blue) and female (red) subjects are depicted individually and the dashed lines demarcate different grading scales for male (blue) and female (red) subjects at Mahidol Hospital for Tropical Diseases. The green line is the 40 year old female subject with elevated AST and ALT values following co-administration of ivermectin plus dihydroartemisinin-piperaquine, depicted in greater detail in Figure 1.

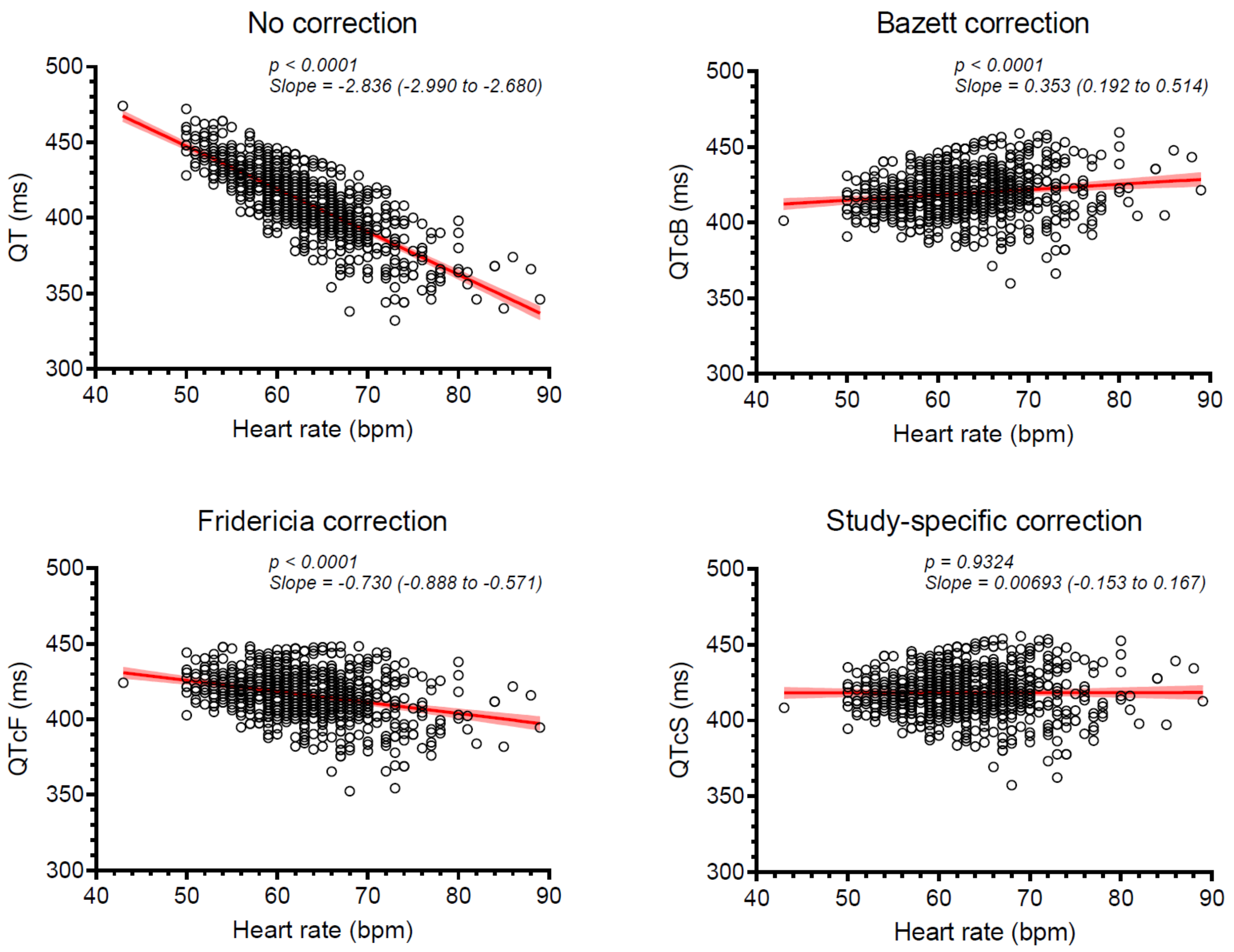

Figure S2. Evaluation of different heart rate-correction factors for electrocardiographic data.

Solid red lines represent mean linear regression of QT-intervals and the heart rates, and shaded areas represent the 95% confidence intervals associated with these estimated mean slopes. bpm represents beats per minute.

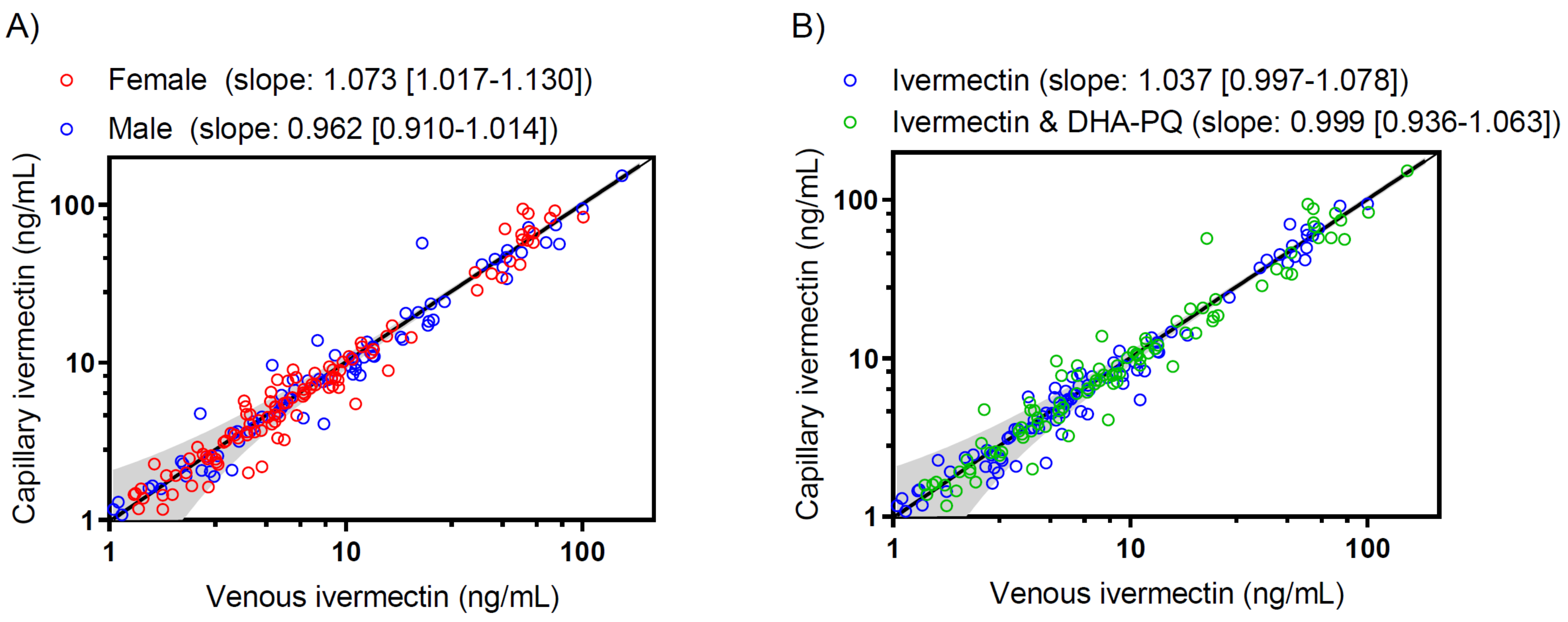

Figure S3. Relationship between venous and capillary blood ivermectin concentrations, stratified for male and female volunteers (A) and treatment regimens (B).

Open circles represent observed venous and capillary blood ivermectin concentrations. Solid black lines represent the overall mean regression line for all data and gray shaded areas represent the 95% confidence interval associated with this slope. Stratified mean slopes [95% confidence intervals] for female (red) and male (blue) volunteers and volunteers receiving ivermectin alone (blue) and ivermectin plus dihydroartemisinin-piperaquine (DHA-PQP) (green) are shown above.

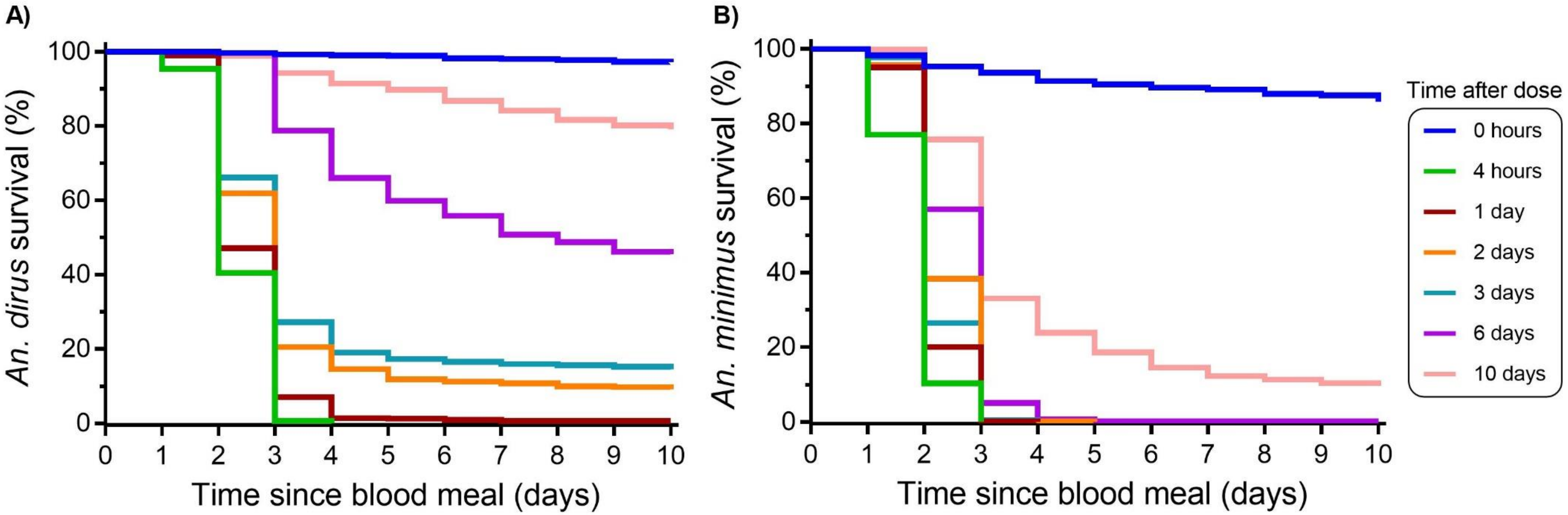

Figure S4. Survival of *Anopheles dirus* (A) and *Anopheles minimus* (B) following ingestion of ivermectin-treated blood from human subjects.

Percent survival of *An. dirus* (A) and *An. minimus* (B) are depicted relative to time of blood meal ingestion. Subjects had blood collected at multiple time points after drug administration and fed to mosquitoes as indicated in the legend.

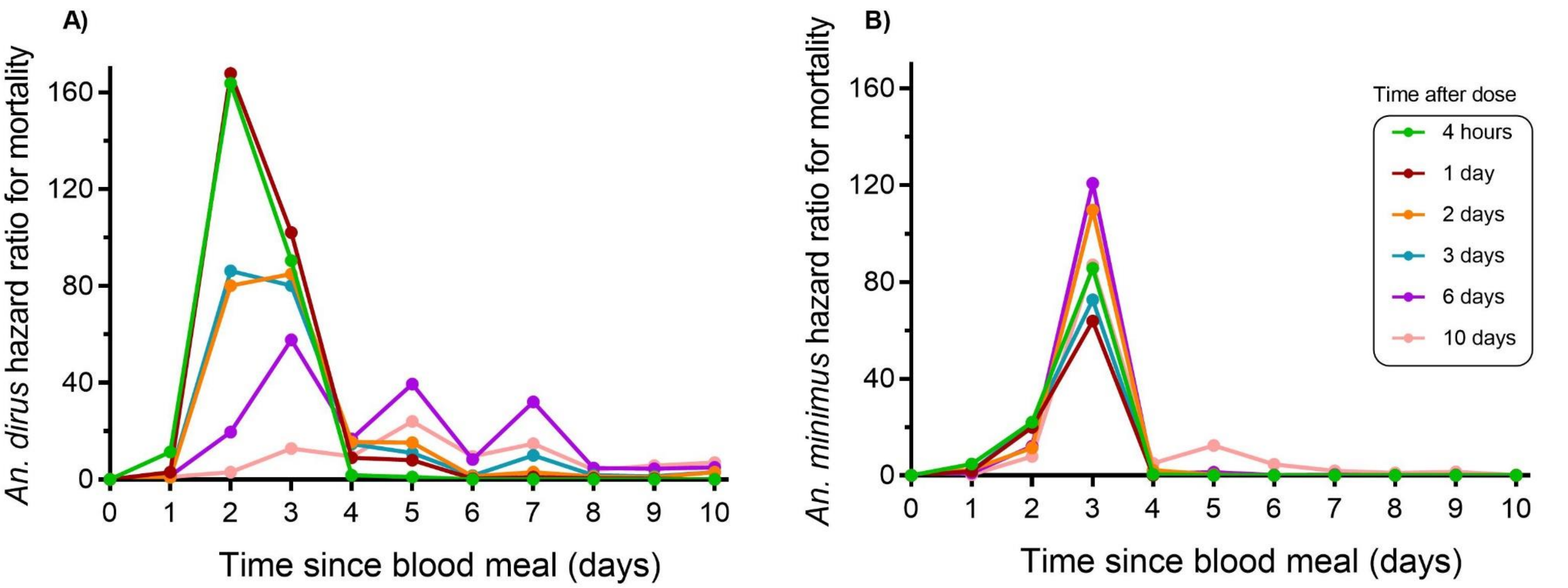

Figure S5. Mosquito hazard ratios for mortality by day post blood meal for *Anopheles dirus* (A) and *Anopheles minimus* (B). Hazard ratios for mosquito mortality, calculated at each day post-blood meal using Poisson regression analysis. Almost all mosquito mortality for both *An. dirus* and *An. minimus* occurs within 3 days post blood ingestion.

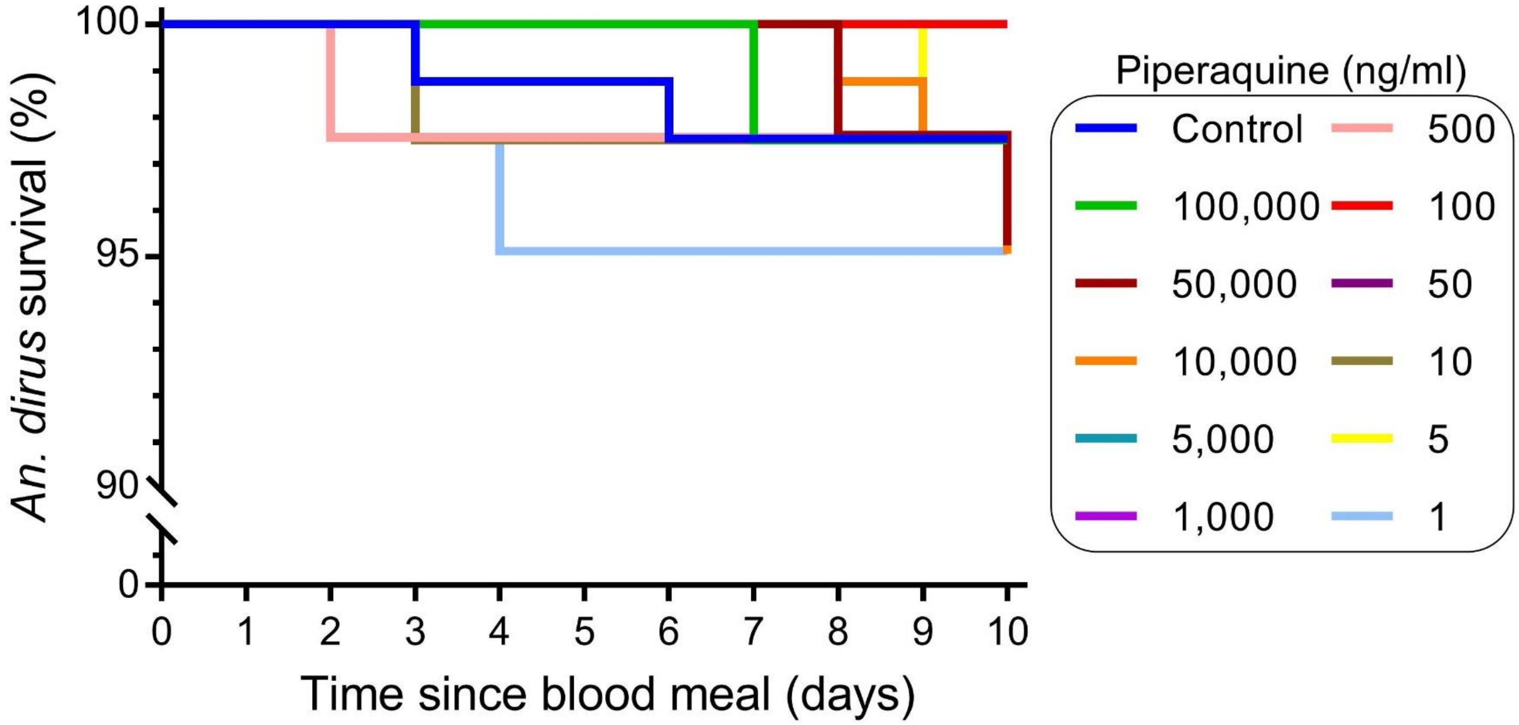

Figure S6. *Anopheles dirus in vitro* mortality response to piperaquine.

Piperaquine-spiked blood was fed to mosquitoes as indicated in the legend with percent survival of *An. dirus* depicted relative to time since blood meal ingestion.

Table S1. Adverse events summary, stratified by treatment regimen.

AE Class	IVM	DHA-PQP	PQ	DHA-PQP +PQ	IVM+PQ	IVM+DHA-PQP	IVM+DHA-PQP+PQ	Total
Neurological	1	2	1	3	2	1	2	12
Gastrointestinal	-	2	1	1	-	1	1	6
Hepatobiliary	-	-	-	-	1	6[a]	3	10
Musculoskeletal	-	-	-	-	2	-	1	3
Allergy	2	-	1	1	1	2	2	9
Infection	1[b]	-	-	3	3	4	2	13
Others	-	-	1	3	2	1	1	8
**Total**	4	4	4	11	11	15	12	61

Data are shown as number of adverse events (AEs) for each organ classification, stratified by treatment regimen. IVM is ivermectin, DHA-PQP is dihydroartemisinin-piperaquine, and PQ is primaquine.

[a] Female subject with AST grade IV which was considered a SAE.

[b] Male subject who was hospitalized due to dengue hemorrhagic fever.

Table S2. Summary of pharmacokinetic drug-drug interactions stratified by treatment regimen.

		Relative difference (90% confidence interval)		
		C_{max}	AUC_T	T_{max}
Ivermectin (IVM)	IVM+DHA+PQP	27.3% (8.61, 49.3)*	33.1% (8.44, 63.3)*	-9.36% (-23.0, 6.64)
	IVM+PQ	8.43% (-0.54, 18.2)	8.26% (-1.93, 19.5)	-14.7% (-24.5, -3.62)
	IVM+DHA+PQP+PQ	31.9% (20.7, 44.2)*	54.4% (35.6, 75.8)*	1.38% (-9.81, 14.0)
Piperaquine (PQP)	PQP+IVM	61.1% (23.1, 111)*	18.1% (-16.7, 67.4)	-0.73% (-14.9, 15.7)
	PQP+PQ	-13.0% (-38.4, 22.9)	-7.96% (-25.5, 13.7)	4.02% (-16.2, 29.2)
	PQP+IVM+PQ	40.3% (2.23, 92.6)*	26.9% (9.03, 47.7)*	-5.95% (-18.9, 9.13)
Dihydroartemisinin (DHA)	DHA+IVM	-10.7% (-26.9, 8.94)	-3.38% (-14.4, 9.02)	-9.27% (-31.2, 19.6)
	DHA+PQ	-21.9% (-37.2, -2.94)	-20.1% (-32.9, -4.80)	17.7% (-10.7, 55.0)
	DHA+IVM + PQ	-4.35% (-14.7, 7.29)	-2.33% (-8.59, 4.35)	0.739% (-19.0, 25.3)
Primaquine (PQ)	PQ+IVM	0.0% (-14.9, 17.5)	-6.35% (-18.8, 7.95)	-30.5% (-45.4, -11.6)*
	PQ+DHA+PQP	16.7% (4.76, 29.9)	2.42% (-6.79, 12.6)	-11.5% (-29.5, 11.0)
	PQ+IVM+DHA+PQP	25.0% (6.61, 46.6)	7.34% (-5.38, 21.7)	-12.8% (-34.0, 15.2)

C_{max} is the maximum concentration, AUC_T is the area under the concentration-time curve from time zero to the last measured concentration, and T_{max} is the time to reach maximum concentration.

* Drug-drug interactions resulting in a clinically significant pharmacokinetic difference of >25%.

Table S3. Pharmacokinetic parameters of ivermectin, primaquine, dihydroartemisinin and piperaquine, stratified by treatment regimen.

**Drug parameter**	**Drug regimen**			
Ivermectin	***IVM***	***IVM+PQ***	***IVM+DHA-PQP***	***IVM+DHA-PQP+PQ***
AUC_T (h×ng/mL)	1,360 (674-3,080)	1,450 (644-2,990)	1,800 (1,030-5,140)	2,180 (1,270-4,210)
T_{max} (h)	6.00 (3.00-6.25)	4.00 (4.00-6.00)	5.00 (3.00-6.00)	6.00 (4.00-6.00)
C_{max} (ng/mL)	56.8 (36.4-119)	60.6 (34.9-101)	68.3 (38.8-188)	78.4 (40.5-137)
Primaquine	***PQ***	***PQ+DHA-PQP***	***PQ+IVM***	***PQ+DHA-PQP+IVM***
AUC_T (h×ng/mL)	1,130 (534-1,620)	1,160 (383-1,660)	944 (442-2,210)	1,090 (580-1,540)
T_{max} (h)	2.50 (1.00-6.00)	3.00 (1.00-4.00)	1.50 (1.33-3.00)	2.00 (1.00-4.00)
C_{max} (ng/mL)	121 (74.8-203)	144 (84.5-244)	109 (68.8-210)	152 (103-256)
Dihydroartemisinin	***DHA-PQP***	***DHA-PQP + PQ***	***DHA-PQP + IVM***	***DHA-PQP+IVM+PQ***
AUC_T (h×ng/mL)	1,000 (620-1,640)	788 (279-1,600)	944 (536-1,900)	929 (655-1,440)
T_{max} (h)	1.50 (1.00-3.00)	2.00 (0.50-4.00)	1.50 (0.50-3.00)	1.50 (0.50-3.00)
C_{max} (ng/mL)	421 (198-678)	283 (166-712)	386 (166-964)	412 (254-713)
Piperaquine	***DHA-PQP***	***DHA-PQP + PQ***	***DHA-PQP + IVM***	***DHA-PQP+IVM+PQ***
AUC_T (h×ng/mL)	17,700 (5,040-26,300)	16,100 (2,370-30,100)	20,500 (4,010-32,700)	23,600 (3,290-34,600)
T_{max} (h)	4.00 (2.00-6.00)	4.00 (1.50-8.00)	4.00 (2.00-6.00)	3.50 (3.00-4.00)
C_{max} (ng/mL)	309 (134-1,040)	321 (76.6-908)	557 (321-1,130)	496 (148-956)

Table S3 illustrates the pharmacokinetic parameters for each compound separated by drug regimen. IVM represents ivermectin, PQ represents primaquine and DHA-PQP represents dihydroartemisinin-piperaquine. Values are presented as median (minimal value-maximal value). AUC_T is the total exposure, measured as area under the concentration-time curve, up until the last observation, C_{max} is the maximum concentration, and T_{max} is the time to reach the maximum concentration.

Text S1. Materials and Methods

Study design and Ethics. The study was conducted at the Hospital of Tropical Medicine, Faculty of Tropical Medicine, Mahidol University in Bangkok, Thailand. The study protocol was approved by the ethics committees of the Faculty of Tropical Medicine, Mahidol University (reference number TMEC 15–004, approval number MUTM 2015-016-02), by the Oxford University Tropical Research Ethics Committee (OXTREC 4–15), and the Walter Reed Army Institute of Research (WRAIR#2228). The trial was registered at ClinicalTrials.gov number NCT02568098. Each volunteer was provided with an explanation of the study and signed a written informed consent before study entry.

Subjects. The inclusion criteria were clinically healthy subjects as judged by a physician, weight between 36 and 75 kg, normal baseline screening results for hematology, biochemistry, urinalysis, and electrocardiogram (ECG), with a QTcF interval of <450 ms and willing to comply with study protocol. Exclusion criteria included a history of drug allergy, alcohol or substance abuse, concomitant medication intake, G6PD deficiency as detected by Beutler's dye test, transaminase enzyme >1.5 times of upper limit of normal, estimated creatinine clearance < 70 ml/min by using Cockcroft-Gault equation, abnormal methemoglobin level (>3 g/dL) or positive HIV, hepatitis B, or hepatitis C serology. Subjects must not have taken any antimalarial drugs within 12 months before study and have no travel history to West or Central Africa to exclude the possibility of *Loa loa* infection. Female subjects had to have a negative serum pregnancy test. Both male and female subject agreed to use effective contraceptive

methods during the study. Some of the volunteers participated in previous studies for dihydroartemisinin-piperaquine (8 volunteers), primaquine (8 volunteers), and their combination (5 volunteers) so their previous safety and pharmacokinetic data were used here (NCT02192944).[1,2]

Study administration, procedures and drugs. The subjects were admitted to the Clinical Therapeutics Unit at the Hospital for Tropical Diseases. The washout periods between doses were >2 weeks after primaquine alone, >4 weeks after ivermectin containing regimen without dihydroartemisinin-piperaquine, and >8 weeks after any regimen containing dihydroartemisinin-piperaquine. Subjects were given a light standard meal (200 kcal with 8 g fat) 30 min before each drug dose and were not allowed to eat within 4 hours after administration of the study drug. Study drugs were taken orally with a glass of water, and water and/or soft drinks without caffeine were permitted 2 hours post-dose. History questionnaire, physical examination and safety laboratory (full blood count, biochemistry measurements, urinalysis) were performed before giving each regimen and at 24 hours post-dose (before discharge). A urine pregnancy test was performed at every admission, before drug administration. Vital signs were checked every 4 hours after dosing. Electrocardiograms were recorded at 0, 1, 2, 4, 8, 12, and 24 h post-dose in each admission.

Methemoglobin was measured at each pharmacokinetic blood sampling time using a noninvasive monitoring machine (Masimo pulse oximeter; SpMet, Japan). Adverse events were captured and graded according to the Division of AIDS (DAIDS) table for grading the severity of adult and pediatric adverse events.[3]

Ivermectin (Vermectin®, Atlantic Laboratories Corp., LTD, Bangkok, Thailand) was administered as 6 mg tablets to the nearest half tablet at a dose of 400 µg/kg. A previous pharmacokinetic model linked to *in vitro* mosquito mortality results indicated ivermectin at 400 µg/kg is the minimal dose that should be used to target *An. dirus*.[4] Dihydroartemisinin-piperaquine (Eurartesim®, Sigma-Tau Industrie Farmaceutiche Riunite S.p.A., Rome, Italy) was administered as three tablets, consisting of 40 mg dihydroartemisinin and 320 mg piperaquine phosphate, for a final single dose of 120 mg/960 mg. Primaquine (primaquine phosphate, Government Pharmaceutical Organization, Bangkok, Thailand) was administered as two 15 mg tablets for a final dose of 30 mg. All drugs were purchased in Thailand.

Sample size. A previous trial demonstrated that the administration of dihydroartemisinin-piperaquine prolonged the QTc interval (baseline mean 420 ms (SD 13.7 ms)) maximally at 4 hours post-exposure, with a mean of 10 ms (SD 13 ms).[1] A further prolongation of an additional 15 milliseconds would lead to a mean QTc of 445 ms, and a one-sided test showed that a sample size of at least 13 subjects would have 90% power to detect such a difference ($\alpha = 0.05\%$). To allow for drop out/lost to follow up, 16 healthy volunteers were enrolled. Power calculations were estimated using PASS v15 (NCSS Statistical Software, Kaysville, UT, USA).

Safety analysis. The safety and tolerability of ivermectin, dihydroartemisinin-piperaquine, and primaquine were assessed by using the Wilcoxon matched-pair signed rank test for continuous variables or McNemar's exact test for categorical variables when drugs were given alone or in combination. The frequencies (%) of adverse events

and serious adverse events, with particular attention to those of potential clinical concern, were presented by treatment group and reported by visit so that any effect of ivermectin, dihydroartemisinin-piperaquine, and primaquine could be assessed. Rise in liver function test parameters were assessed at baseline (hour 0) to 24 hours post administration and compared between treatment groups by an analysis of covariance (ANCOVA), analyzed with STATA v15 (StataCorp, College Station, TX, USA). Tests of significance were performed at 5% significance level.

Pharmacokinetic sampling. For the pharmacokinetic assessment of primaquine, dihydroartemisinin, and piperaquine venous blood samples (2 ml) were collected into fluoride-oxalate tubes at 0 (predose), 0.5, 1, 1.5, 2, 3, 4, 6, 8, 12, and 24 hours, and on day 2. An indwelling catheter was used for the multiple serial blood collections from 0 to 12 hours postdose. Additional blood samples were taken for piperaquine measurements on days 3, 6, 10, 14, 21, and 35. After collection, blood samples were centrifuged for 7 min at 2,000 *g* at 4°C, and plasma was stored at -70°C or lower. For pharmacokinetic assessment of ivermectin venous blood samples (0.5 ml) were collected into sodium heparin tubes at 0 (predose), 0.5, 1, 1.5, 2, 3, 4, 6, 8, 12, and 24 hours and on days 2, 3, 6, and 10. Capillary blood samples (0.2 ml) were collected in sodium heparin capillary tubes at 0 (predose), 4, and 24 hours and on days 2, 3, 6, and 10 for ivermectin measurement. Ivermectin samples were stored as whole blood at -70°C or lower. All samples were transferred to the Department of Clinical Pharmacology, Mahidol-Oxford Tropical Medicine Research Unit, Bangkok, Thailand, for drug measurements. The laboratory participates in the WorldWide Antimalarial Resistance Network (WWARN)

quality control and assurance proficiency testing program[5] with satisfactory performance (http://www.wwarn.org/toolkit/qaqc).

Drug analysis. Drug extraction was performed using solid-phase extraction. The limit of quantification was 1.57 ng/ml for dihydroartemisinin, 1.20 ng/ml for piperaquine, 0.912 ng/ml for primaquine, and 0.776 ng/ml for ivermectin. Three replicates of quality control samples at low, middle, and high concentrations were analyzed within each batch of clinical samples to ensure precision and accuracy during drug measurements. The total precision (*i.e.*, relative standard deviation [SD]) for all drug measurements was below 10% during drug quantification.

Pharmacokinetic analysis. Individual subject drug concentration-time data were evaluated using a non-compartmental approach as implemented in Phoenix v8.1 (Certara, Princeton, NJ, USA). Visual inspection of all concentration-time profiles were performed to identify any errors in the raw data. Total exposure up to the last measured concentration (AUC_T) was calculated using the linear trapezoidal method for ascending concentrations and the logarithmic trapezoidal method for descending concentrations. The maximum drug concentration (C_{max}) and time to maximum concentration (T_{max}) were taken directly from the observed data. Samples were collected for up to 24 hours only in three volunteers receiving dihydroartemisinin-piperaquine alone. For these individuals, AUC calculations were truncated at 24 hours for all piperaquine-containing regimens to not bias the results. Differences in pharmacokinetics parameters between males and females were evaluated with a Mann-Whitney U-test.

Pharmacokinetic parameter estimates from the different regimens were analyzed with the bioequivalence function in Phoenix using log-transformed pharmacokinetic exposure parameters (*i.e.* C_{max} and AUC_T) and time to maximum concentration (T_{max}), to assess the exposure of the drug when administered alone versus that in combination. Drug-drug interactions were assessed by calculating the point estimate of the geometric mean ratio and the 90% confidence interval around this estimate. The results were visualized in GraphPad Prism v7.2 (GraphPad Software, San Diego, CA, USA).

QT-interval normalization. The commonly used Fridericia-correction (QTcF; correction factor (α) = 1/3) and Bazett-correction (QTcB; α = 1/2) were used to normalize measured QT-intervals with heart rate (Eq. 1 & 2).

$$QTc = \frac{QT}{RR^{\alpha}} \qquad \text{Equation 1}$$

$$RR = \frac{60}{Heart\ rate} \qquad \text{Equation 2}$$

It is well known that these are not always optimal for a particular data set and an optimal correction factor was calculated using all available QT and associated heart rate data (Eq. 3).

$$\ln(QT) = \alpha \times \ln(RR) + intercept \qquad \text{Equation 3}$$

Uncorrected and corrected QT-intervals (QTcF, QTcB and QTcS) were plotted against individual heart rates to assess the appropriateness of the evaluated correction factor. Any correction factor that did not result in a significant residual trend was assumed to appropriately correct this data. Corrected QT-intervals (QTc) were used to evaluate the potential electrocardiographic impact of evaluated drugs and combinations. Individually calculated differences between baseline QTc and observed QTc after drug

administration (ΔQTc) was used to evaluate the QT-interval prolonging properties of drug combinations. A paired ANOVA-test was used to evaluate statistical differences between drug regimens. Furthermore, individual ΔQTc were also plotted against corresponding drug concentrations and ordinary linear regression was used to quantify the magnitude of potential QT-interval prolongations. All QT data were analyzed and visualized using GraphPad Prism.

Mosquitoes, experimental design, and survival analyses. All mosquitoes were reared at the Armed Forces Research Institute of Medical Sciences, Department of Entomology in Bangkok, Thailand. Adult mosquitoes used for experiments were provided 10% sucrose solution *ad libitum*. Mosquitoes were reared at 25 ± 2°C and 80 ± 10% relative humidity, and 12 hours light:12 hours dark photoperiod. Mosquitoes were between 5 and 8 days post emergence at time of blood feed, and mosquitoes were sugar starved with access to water from 12 to 18 hours prior to their first blood meal.

Whole blood samples were collected in sodium heparin tubes at 0, 4, and 24 hours, and on days 2, 3, 6, and 10 from all drug regimens that contained ivermectin. At each time point approximately 600 µl of whole blood was fed via membrane feeders at 37°C to groups of 100 *An. dirus* and 100 *An. minimus*. Up to 40 mosquitoes of each species were gently transferred via aspiration to clean cardboard containers (0.5 L). Mosquitoes were maintained in an incubator at 25 ± 1°C and 80 ± 10% humidity, and offered 10% sucrose *ad libitum*. Mosquito survival was monitored daily for ten days and any dead mosquitoes were removed by aspiration and recorded. At day 10 post blood meal any remaining mosquitoes were frozen and recorded as alive.

Piperaquine compound was dissolved in 0.5% lactic acid to 10 mg/ml and serially diluted in distilled water, then 10 µl was added to 990 µl of whole blood to achieve a range of concentrations from 1 – 100,000 ng/ml. Controls consisted of 0.5% lactic acid diluted in distilled water to match the highest concentration of piperaquine fed to mosquitoes. Blood meals were then fed to mosquitoes via membrane feeders. Mosquitoes were maintained in the same incubator as trial mosquitoes and their mortality was observed daily for ten days, any remaining mosquitoes at day 10 were frozen and counted as alive. Two replicates with *An. dirus* (n = 567) were performed.

Survival curves at each time point were compared to baseline within each treatment regimen using Log-Rank survival curve analysis (Mantel-Cox method). Cumulative mosquito mortality at day 10 between treatment groups were assessed at each time point using a Z-test for proportions. The lethal concentration that kills 50% of mosquitoes (LC_{50}) were estimated using a normalized concentration-response analysis (IC_{50} and Hill). All mosquito survival analyses performed with GraphPad Prism. Hazard ratios for mosquito mortality, calculated at each day post-blood meal using Poisson regression analysis using STATA. There were several instances when no mosquitoes died in the control group at several days post-feeding within a regimen, therefore, hazard ratios could only be calculated using data from all four drug regimens combined.

Additional References:

1 Hanboonkunupakarn, B. *et al.* Open-label crossover study of primaquine and dihydroartemisinin-piperaquine pharmacokinetics in healthy adult Thai subjects. *Antimicrobial Agents and Chemotherapy* **58**, 7340-6 (2014).

2 Jittamala, P. *et al.* Pharmacokinetic interactions between primaquine and pyronaridine-artesunate in healthy adult Thai subjects. *Antimicrobial Agents and Chemotherapy* **59**, 505-13 (2015).

3 National Institute of Allergy and Infectious Diseases. Division of AIDS (DAIDS) table for grading the severity of adult and pediatric adverse events. Vol. Version 2.0 (ed. National Institutes of Health) 30 (2014).
4 Kobylinski, K. *et al.* Ivermectin susceptibility and sporontocidal effect in Greater Mekong Subregion *Anopheles Malaria Journal* **16**, e280 (2017).
5 Lourens, C. *et al.* Benefits of a pharmacology antimalarial reference standard and proficiency testing program provided by the Worldwide Antimalarial Resistance Network (WWARN). **58**, 3889-94 (2014).
